# Validation of UAV-based alfalfa biomass predictability using photogrammetry with fully automatic plot segmentation

**DOI:** 10.1038/s41598-021-82797-x

**Published:** 2021-02-08

**Authors:** Zhou Tang, Atit Parajuli, Chunpeng James Chen, Yang Hu, Samuel Revolinski, Cesar Augusto Medina, Sen Lin, Zhiwu Zhang, Long-Xi Yu

**Affiliations:** 1 Department of Crop and Soil Sciences, Washington State University, Pullman, WA USA; 2 United States Department of Agriculture-Agricultural Research Service, Plant Germplasm Introduction and Testing Research, 24106 N Bunn Road, Prosser, WA 99350 USA

**Keywords:** Plant breeding, Plant genetics

## Abstract

Alfalfa is the most widely cultivated forage legume, with approximately 30 million hectares planted worldwide. Genetic improvements in alfalfa have been highly successful in developing cultivars with exceptional winter hardiness and disease resistance traits. However, genetic improvements have been limited for complex economically important traits such as biomass. One of the major bottlenecks is the labor-intensive phenotyping burden for biomass selection. In this study, we employed two alfalfa fields to pave a path to overcome the challenge by using UAV images with fully automatic field plot segmentation for high-throughput phenotyping. The first field was used to develop the prediction model and the second field to validate the predictions. The first and second fields had 808 and 1025 plots, respectively. The first field had three harvests with biomass measured in May, July, and September of 2019. The second had one harvest with biomass measured in September of 2019. These two fields were imaged one day before harvesting with a DJI Phantom 4 pro UAV carrying an additional Sentera multispectral camera. Alfalfa plot images were extracted by GRID software to quantify vegetative area based on the Normalized Difference Vegetation Index. The prediction model developed from the first field explained 50–70% (R Square) of biomass variation in the second field by incorporating four features from UAV images: vegetative area, plant height, Normalized Green–Red Difference Index, and Normalized Difference Red Edge Index. This result suggests that UAV-based, high-throughput phenotyping could be used to improve the efficiency of the biomass selection process in alfalfa breeding programs.

## Introduction

Alfalfa is the most widely cultivated forage legume, with approximately 30 million hectares planted worldwide^1^. In 2018, the United States produced 52 million tons of alfalfa hay, valued at around $8 billion^2^. Alfalfa’s high nutritional value, including 15–22% crude protein and an abundance of vitamins and minerals^3^, makes it well-suited for animal feed. Alfalfa also brings long-term ecological benefits to society^4,5^. For example, alfalfa plays a significant role in improving soil fertility because it naturally forms symbiotic associations with soil bacteria, such as *Sinorhizobium meliloti*, to fix atmospheric nitrogen. As a result, the soil’s nitrogen content is improved for future crops. In addition, the perennial nature of the crop, along with its deep root system (up to 15 m), helps prevent soil erosion^5,6^.

Alfalfa breeders have successfully improved simple genetic traits, such as winter hardiness and disease and pest resistance, that are controlled by major genes^7–9^. However, genetic improvements in complex traits such as forage biomass and seed yield have lagged behind other annual crops^7,10^. Because biomass has low heritability^11^, low predictability^12^, and is controlled by a combination of multiple genes and their interactions with environmental factors, breeding with high-selection intensity is critical for realizing genetic improvements^13–16^. Current alfalfa breeding programs are primarily based on phenotypic selection, which involves intensive time and labor to conduct manual field screening of breeding populations^17^. Furthermore, implementing crop trials across multiple environments so that the extent of genotype-by-environment interactions can be measured to evaluate trait stability across locations is both cost and labor prohibitive^18^. The perennial nature of alfalfa and its capacity for multiple harvests increases the intensity of phenotyping. This phenotyping burden for biomass has been the major bottleneck for genetic improvement in alfalfa^19^.

Unmanned Aerial Vehicles (UAVs) are efficient phenotyping platforms that can be equipped with different sensors to provide accurate and rapid phenotypic information related to various crops. RGB imagery from digital cameras achieved an accurate estimation (R^2^ = 0.96) of potato crop emergence rate^20^. Different Vegetation Indices (VIs), such as the Normalized Difference Vegetation Index (NDVI), derived from UAV-based multispectral imagery showed different sensitivities for wheat yellow rust at different stages^21^. In rice, VIs from UAV-based hyperspectral imagery achieved yield estimations with higher accuracies using time-series than using single stage^22^. In cotton, a UAV equipped with a thermal sensor was operated to monitor water stress^23^. For the estimation of biomass, UAV-LiDAR achieved higher accuracy in sugar beet and winter wheat than in potato^24^. The 3D point cloud-based methods were also tested in grassland biomass estimation. The crop surface height (CSH), calculated with 3D point clouds using the structure from motion (SfM) of UAV images, achieved an average normalized root mean square error (nRMSE) of 19.5% for the dry biomass estimation among multiple harvest times and grasslands^25^. Multiple linear regression models and machine learning models could attain high accuracy for maize above-ground biomass estimation^26^ using statistical variables related to crop height derived from SfM data.

In recent years, more research has been carried out to predict crop biomass using VIs constructed from different spectrums. In sugarcane, the Green–Red Vegetation Index (GRVI) can predict crop yield well (R^2^ = 0.69)^27^. In rice, a high accuracy (R^2^ = 0.8) was achieved for biomass prediction when including 35 VIs captured from UAV multispectral imagery in the LASSO regression mode^28^. By combining plant height information with VIs, such as NDVI, Enhanced Vegetation Index (EVI), and Ratio Vegetation Index (RVI), a prediction accuracy of R^2^ = 0.74 can be achieved using partial least squares regression for above-ground biomass of winter wheat^29^. In grass swards, a Pearson correlation coefficient of 0.98 was achieved between observed and predicted dry matter with the combination of a canopy height model, RGB images, and VIs^30^. In black oat, accurate estimations of dry biomass and fresh biomass were also achieved^31^.

In alfalfa, a prediction accuracy of R^2^ = 0.64 was achieved when the prediction model included canopy height, which was measured using a ground-based mobile sensing method^32^. UAV-based multispectral imagery also achieved high accuracy (R^2^ = 0.87) with canopy area defined by the Global Positioning System (GPS) coordinates in ArcGIS^33^. Multiple software packages were developed for plot segmentation with uniform spacing, including Progeny (https://www.progenydrone.com), FIELDimageR^34^, and EasyMPE^35^. These software packages work well for plants with canopy layouts that are clearly defined by straight lines. However, for alfalfa field plots, straight lines cannot easily separate all adjacent rows and columns. A software package, Phenalysis, was developed to partially solve this problem^36^. Users can define the field boundary and number of rows and columns to initial plot segments with uniform space. The software can automatically adjust the boundary of individual plots based on canopy coverage. Recently, GRID^37^ was developed to conduct fully automatic extraction of field plots that are not in straight layouts, including initiation segmentation without asking for number of rows and columns and exclusion of non-vegetative areas.

In this study, we used GRID to conduct fully automatic field plot segmentation for alfalfa biomass prediction. In 2019, we harvested two alfalfa fields in Prosser, Washington, USA. Biomass was weighed for individual plots on three harvest dates for the first field (planted in 2018) and on one harvest date for the second field (planted in 2017). UAV images were taken 30.48 m above the fields one day before harvesting. With fully automatic segmentation in orthomosaic photos derived from UAV images, the first field was used as the training field to identify features and models. Then, the second field was used as the testing field to validate the model's prediction accuracy and to lend support for using the UAV-based imagery method for broader applications.

## Results

### Weak correlation of manually harvested biomass among months of harvest

The alfalfa harvested in May resulted in much higher biomass than the other cuttings in July and September because of full dormancy during the long winter season (Table 1). The average plot biomass in May weighed 4.28, 3.48, and 3.89 kg for replicates 1, 2, and 3, respectively. Then, as the dry season started, average plot biomass dropped to about one-half of the May values by July and further to about one-eighth by September. For example, in July, average plot biomass measured 2.69, 2.23, and 1.97 kg for replicates 1, 2, and 3, respectively. By September, these biomass values dropped even further to 0.49, 0.28, and 0.34 kg for replicates 1, 2, and 3, respectively. These biomass reductions can also be easily visualized on the UAV images (Figs. S1 and S2). The manually harvested biomass values for each plot are displayed as the actual measurement units (kg) and as color-coded heatmaps in Fig. S3. The plant heights extracted from UAV images for the first field are shown in Fig. S4.

**Table 1:** Statistics of biomass and six image features evaluated in the first alfalfa field.

Month		May			July			September		
Replicate		1	2	3	1	2	3	1	2	3
Biomass (kg)	Avg	4.28	3.48	3.89	2.69	2.23	1.97	0.49	0.28	0.34
	SD	0.58	0.55	0.62	0.91	0.74	0.77	0.35	0.11	0.15
Area pixels /100	Avg	46.29	43.91	44.73	38.41	34.76	35.22	17.18	15.65	15.65
	SD	5.21	6.02	6.16	6.15	5.25	5.94	6.32	3.09	3.17
Plant Height (cm)	Avg	35.62	32.87	33.00	39.81	39.13	37.51	19.63	21.68	16.77
	SD	6.83	6.31	5.82	6.84	5.83	5.39	6.46	7.58	4.25
NDVI × 100	Avg	37.39	38.09	42.22	40.60	39.08	41.19	27.61	28.01	27.05
	SD	5.38	4.89	5.08	4.20	3.32	3.53	6.24	3.88	4.23
NDRE × 100	Avg	− 40.79	− 40.71	− 40.86	− 40.84	− 40.77	− 41.04	− 41.46	− 41.19	− 41.30
	SD	0.50	0.48	0.50	0.48	0.47	0.43	0.53	0.48	0.54
NGRDI × 100	Avg	28.22	28.15	28.47	24.45	23.77	23.97	14.63	14.97	14.31
	SD	1.08	1.20	1.20	1.58	1.52	1.74	2.64	1.55	1.66

Values for observed biomass and each image feature are displayed as the plot average (Avg) and standard deviation (SD) for each combination of month and replicate.

**Figure 1:**
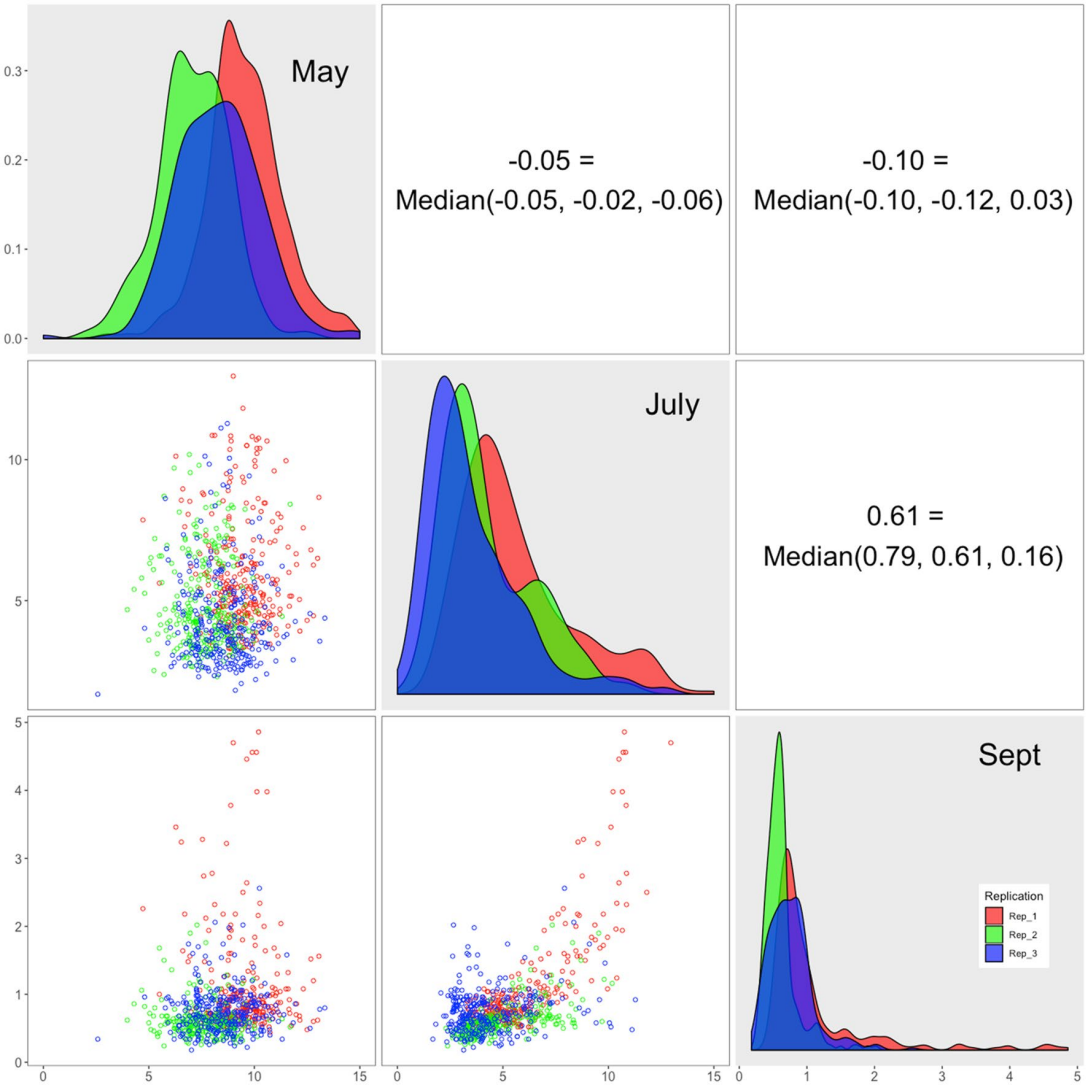
Distributions, scatter plots, and correlations among biomass harvested in May, July, and September of 2019. The distribution of biomass values is displayed on the diagonal for alfalfa plots harvested on May 6–7, July 8–9, and September 3–4, 2019, with replications indicated by colors. Replication 1 and 2 contained 269 plots; replication 3 contained 270 plots. The correlations among harvest months are displayed as scatter plots in the lower triangular area and as the Pearson correlation coefficients in the upper triangular area.

The magnitude of change in the Pearson correlation coefficients for plots differed across the three harvests. Some coefficients changed more than others and even reversed direction (Fig. 1). The biomass in both July and September resulted from drought and high temperature conditions. Thus, the Pearson correlation coefficient between July and September is much higher than the correlation between May and either July or September. The magnitude of the correlation coefficients between biomass values in May and biomass values in either July or September were low (R < 0.12). The Pearson correlation coefficients between July and September were 0.79 and 0.61 for replicates 1 and 2, respectively. The other reason for these results could be the differences in the micro-spatial environments. The control check plots exhibited Pearson correlation coefficients of 0.11 between May and July, 0.24 between May and September, and 0.62 between July and September, suggesting measurements across the months are necessary to evaluate the overall performances.

### Image feature selection

**Figure 2:**
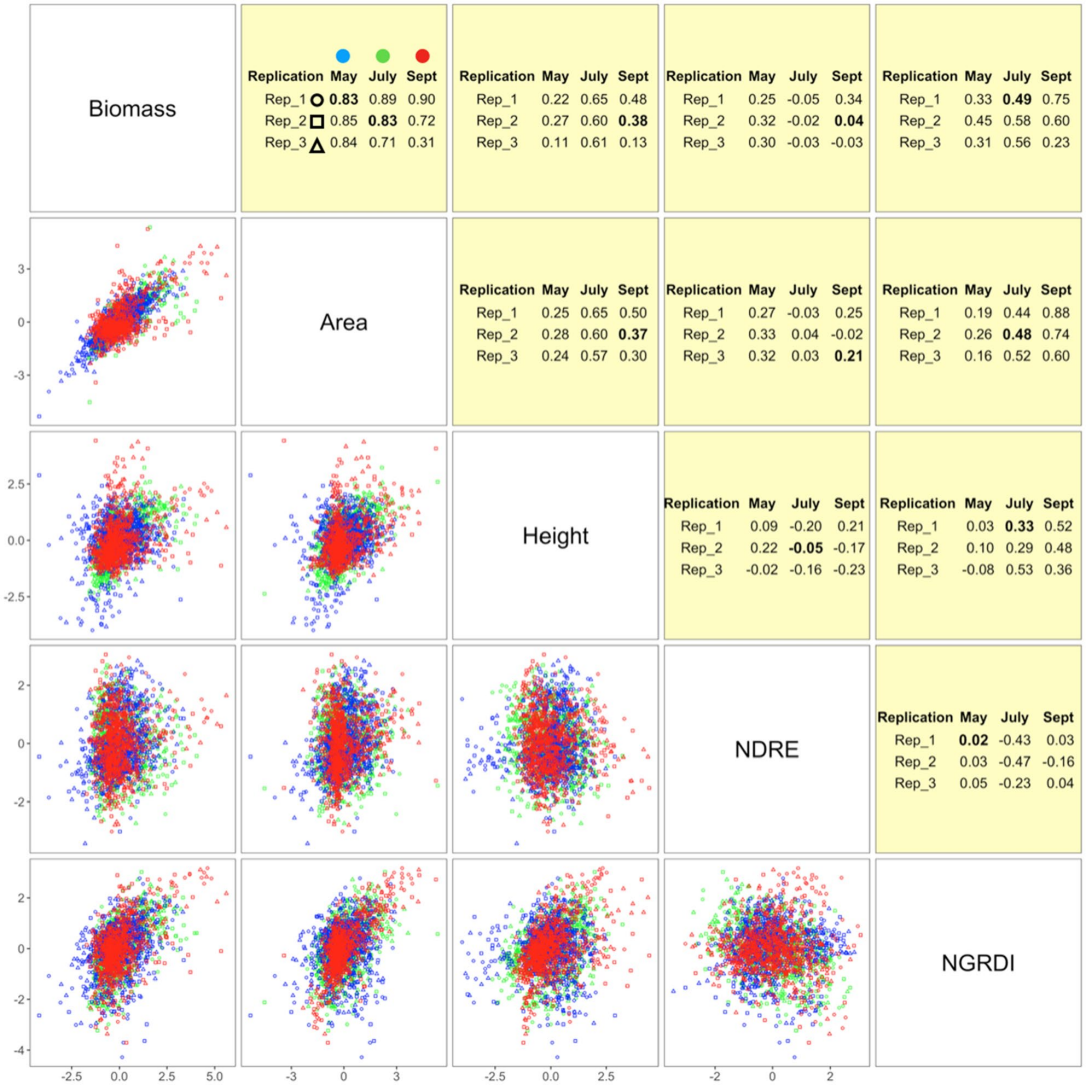
Correlations among biomass and four image features. The correlations of image features with observed biomass are demonstrated as Pearson correlation coefficients in the upper triangular area and scatter plots in the lower triangular area. The four image features are standardized within combinations of month and replicate. The medians of correlation coefficients are highlighted in bold text. Months are illustrated by colors and replicates by shapes. The four image features are canopy area, plant height, Normalized Difference Red Edge (NDRE) Index, and Normalized Green–Red Difference Index (NGRDI).

Among all 22 image features evaluated (Table S1), vegetative area had the strongest correlation with biomass, and the correlation remained stable over the entire growing season. Median correlation coefficients over the three replicates were 0.83, 0.83, and 0.72 for May, July, and September, respectively. The only exception was replicate 3 in September. The biomass of replicate 3 in September had weak correlations with all of the image features (R = 0.31), suggesting potential labor or calculation errors in manually harvested biomass (Fig. 2).

**Figure 3:**
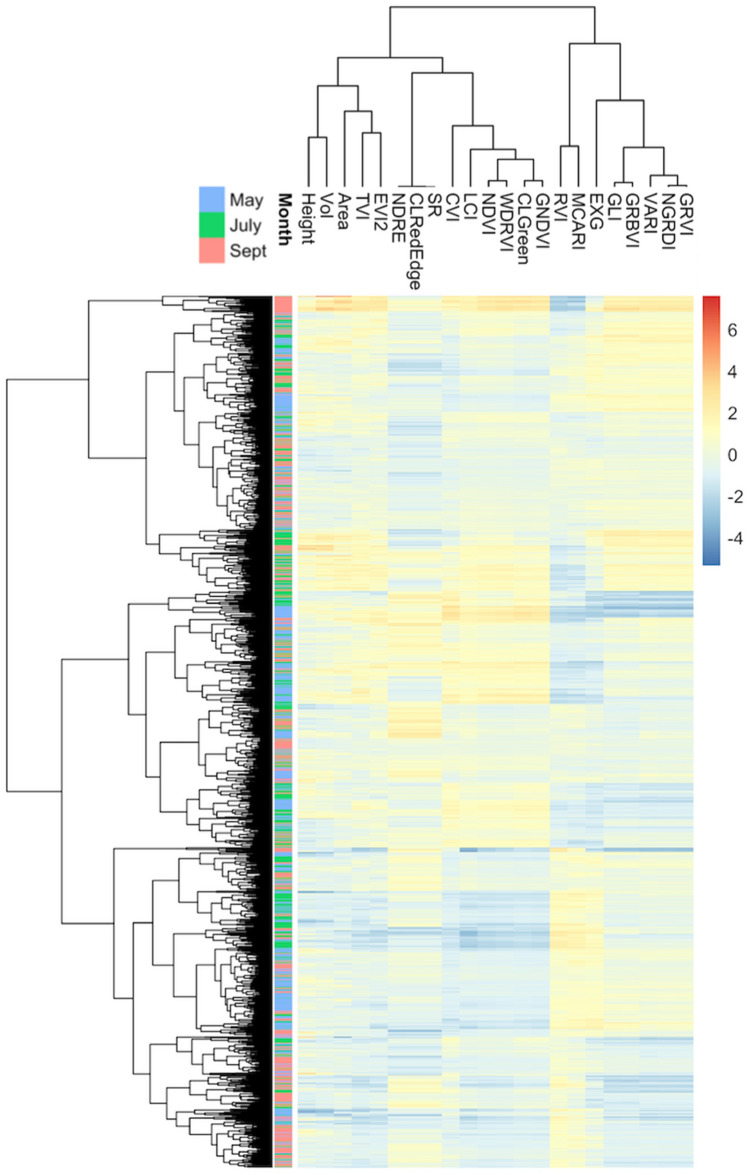
Two-way cluster analysis of plots and image features after standardization. The standardizations were conducted within each combination of month and replicate. The image features include plant area, height, volume, and 19 vegetation indices defined in Table S1.

After vegetative area was determined as the first variable potentially capable of predicting biomass, we selected plant height as the second variable for two reasons. First, volume, defined as the product of area and plant height, was the variable with the second strongest correlation with the biomass (R = 0.66) (Fig. S7). Second, plant height was in a cluster with volume and separated from the other variables (Fig. 3). Plant height was highest in July (median of 39.13 cm) and lowest in September (median of 19.63 cm). Plant height exhibited a moderate correlation (R > 0.6) with biomass in July and a weak correlation in other months (R < 0.5). The correlation between plant height and plant area follows the same trend. Plant height demonstrated relatively stable and moderate-to-low correlations with NDVI, with R ranging from 0.26 to 0.62 among all replicate-month combinations.

During the process of selecting the third variable, we first excluded NDVI and variables positively correlated with NDVI, which was used to define area. NDVI exhibited moderate correlation with area. We also excluded variables that exhibited strong negative correlations with NDVI [e.g., Modified Chlorophyll Absorption in Reflectance Index (MCARI)] and excluded the variables that exhibited weak correlations with biomass [e.g., Excess Green Index (EXG)]. The remaining variables appeared as two clusters (Figs. 3, S8). One cluster contained three variables: Normalized Difference Red Edge Index (NDRE), Simple Ratio (SR), and Chlorophyll Index Red Edge (CI_RedEdge_). These three variables were 100% correlated; we selected NDRE as the representative. Of the variables in the second cluster, we chose Normalized Green–Red Difference Index (NGRDI) because it had the highest correlation with biomass. Therefore, our prediction model contained four variables: Area, Height, NDRE, and NGRDI.

Finally, we evaluated each single variable's contribution to predict biomass by comparing the residual sum of squares (RSS) of the full model (with all variables included) to the RSS of reduced models, each with only one variable excluded (Fig. S9). The degree of increase in the RSS was used as the evaluation criterion. Plant area demonstrated the largest effect for predicting biomass based on the increase in the RSS of the model with all variables except area. Variables that were strongly correlated with area (e.g., CI_Green_) also had a similar effect on predicting biomass.

### Cross-validation within the first field

**Figure 4:**
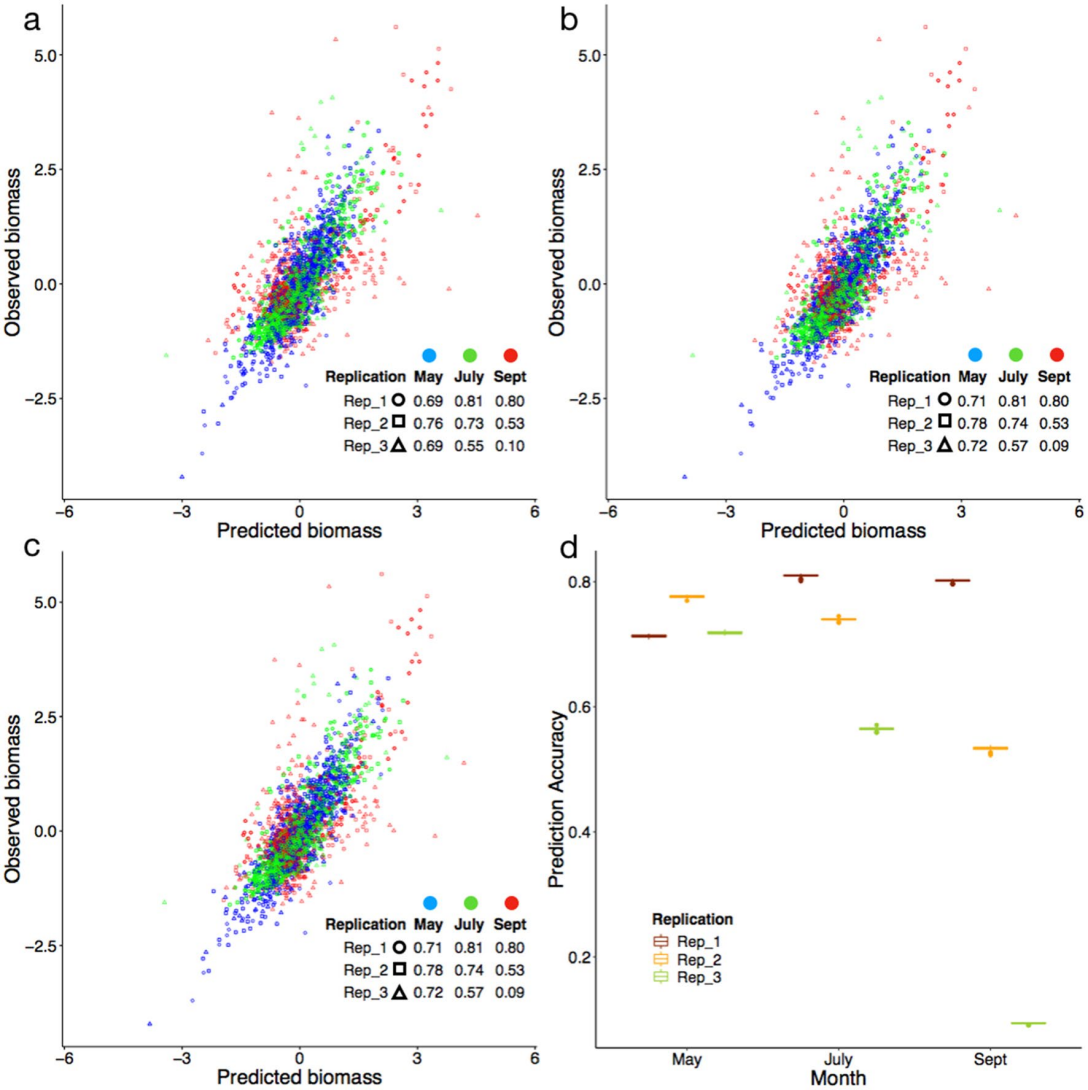
Prediction accuracy under temporal, spatial, and mixed cohort validation. Prediction accuracy was calculated as the coefficient of determination (R^2^). (**a**) The temporal cohort was based on months (May, July, and September). Two months were selected as the training data and the remaining month was used for validation. The selection process was repeated until all months were tested. (**b**) Similarly, two replicates were selected as training under the spatial cohort with the remaining replicate for validation. The selection process was repeated until all replicates were tested. (**c**) Under a mixed cohort, all plots of three replicates and three months were randomly assigned evenly into three folds. Two folds were used for training and the remaining fold for validation. The process was repeated until all folds were tested. The random assignment was iterated 100 times. One of the iterations was arbitrarily selected for illustration. (**d**) The distribution of the prediction accuracy is demonstrated by the box plot.

To examine prediction accuracy with these four features, we conducted cross-validation tests with three schemes, including temporal, spatial, and a mixture of spatial and temporal. These three schemes are three different ways to split training data to implement threefold cross-validation. Table 2 summarizes the regression coefficients of these four variables in models trained with different sets of data. Under the temporal scheme, we chose one month as the test population and hid that month’s observed biomass. The predicted biomass was derived from the UAV images for the test month based on the prediction formula derived from the observed biomass and UAV images from the other two months. The coefficient of determination (R^2^) was calculated between the observed and the predicted biomass for the test month. We iterated month as the test population until all months were tested. The average of the R^2^ across the three testing months was used as the prediction accuracy. Biomass per plot for any month can be predicted with UAV images based on data from other months (Fig. 4a). The median prediction accuracy was R^2^ = 0.69 for all plots within all nine combinations of replicates and months. The outlier combination was replicate 3 in September. The biomass of the plots within this combination was not predictable (R^2^ = 0.10). The prediction results of the first alfalfa field are displayed as values and heat maps in Fig. S5.

**Table 2:** Regression coefficients of prediction models trained with different sets of data.

Training data	Area	Height	NDRE	NGRDI
Full	0.66	0.07	0.04	0.15
May and July	0.72	0.06	0.05	0.20
May and September	0.66	0.02	0.05	0.13
July and September	0.57	0.14	0.04	0.14
Rep 1 and 2	0.71	0.07	0.07	0.17
Rep 1 and 3	0.66	0.06	0.03	0.13
Rep 2 and 3	0.60	0.07	0.03	0.16
Cross-validation (± SD)	0.66 ± 0.01	0.07 ± 0.01	0.04 ± 0.01	0.15 ± 0.01

*SD* standard deviation (100 replicates).

Similarly, under the spatial scheme, we chose one replicate as the test dataset and hid its observed biomass. The predicted biomass was derived from the UAV images of this replicate based on the prediction formula derived from the observed biomass and UAV images from the other two replicates. The R^2^ was calculated between the observed and the predicted biomass for the test replicate. We iterated replicate as the test dataset until all replicates were tested. The average of the R^2^ across the three test replicates was used as the prediction accuracy. Biomass per plot for any replicate can be predicted with UAV images based on data from other replicates (Fig. 4b). The median prediction accuracy was R^2^ = 0.72 for plots within the nine combinations of replicates and months. Again, the outlier combination was replicate 3 in September. The biomass in the plots of this combination was not predictable (R^2^ = 0.09).

Under the mixed scheme, we randomly divided plots into three folds regardless of month or replicate. We chose one-fold as the test dataset and hid its observed biomass. The predicted biomass was derived from the UAV images of this fold based on the prediction formula derived from the other two folds. The R^2^ was calculated between the observed and the predicted biomass for the test fold. We iterated fold as test population until all folds were tested. The average of the R^2^ across the three test folds was used as the prediction accuracy. The random process was iterated 100 times. One iteration was arbitrarily chosen for illustration (Fig. 4c). For this iteration, the median prediction accuracy was R^2^ = 0.72 for all plots within the nine combinations of replicates and months. Again, the outlier was replicate 3 in September. The biomass in the plots of this combination was not predictable (R^2^ = 0.09). The distribution of the prediction accuracies over the 100 iterations is illustrated by the box plot (Fig. 4d) for the nine combinations of months and replicates. The biomass of any one-third of plots can be predicted with UAV images based on the other plots. The median of the average prediction accuracy was R^2^ = 0.72 for plots within the nine combinations of replicates and months. Again, the outlier was replicate 3 in September. The biomass in the plots of this combination was not predictable (R^2^ = 0.09).

### Validation with the second field as an independent experiment

The objective of this study was to develop a high-throughput method to phenotype alfalfa biomass. Therefore, examining whether the method and prediction model developed with data from the first field would work in another field was critical for two reasons. First, alfalfa is a perennial crop. Consequently, biomass will vary not only among harvesting seasons, but also among plants of different ages. The first alfalfa field, planted in 2018, was in its second growing season during our manual harvesting and UAV imaging. We were interested to investigate if our biomass prediction method trained in such a field would accurately predict biomass in a field with older-aged alfalfa. The other reason for this additional validation experiment was to eliminate the possibility of concluding that the experimental results found in the first field were due to model overfitting.

**Figure 5:**
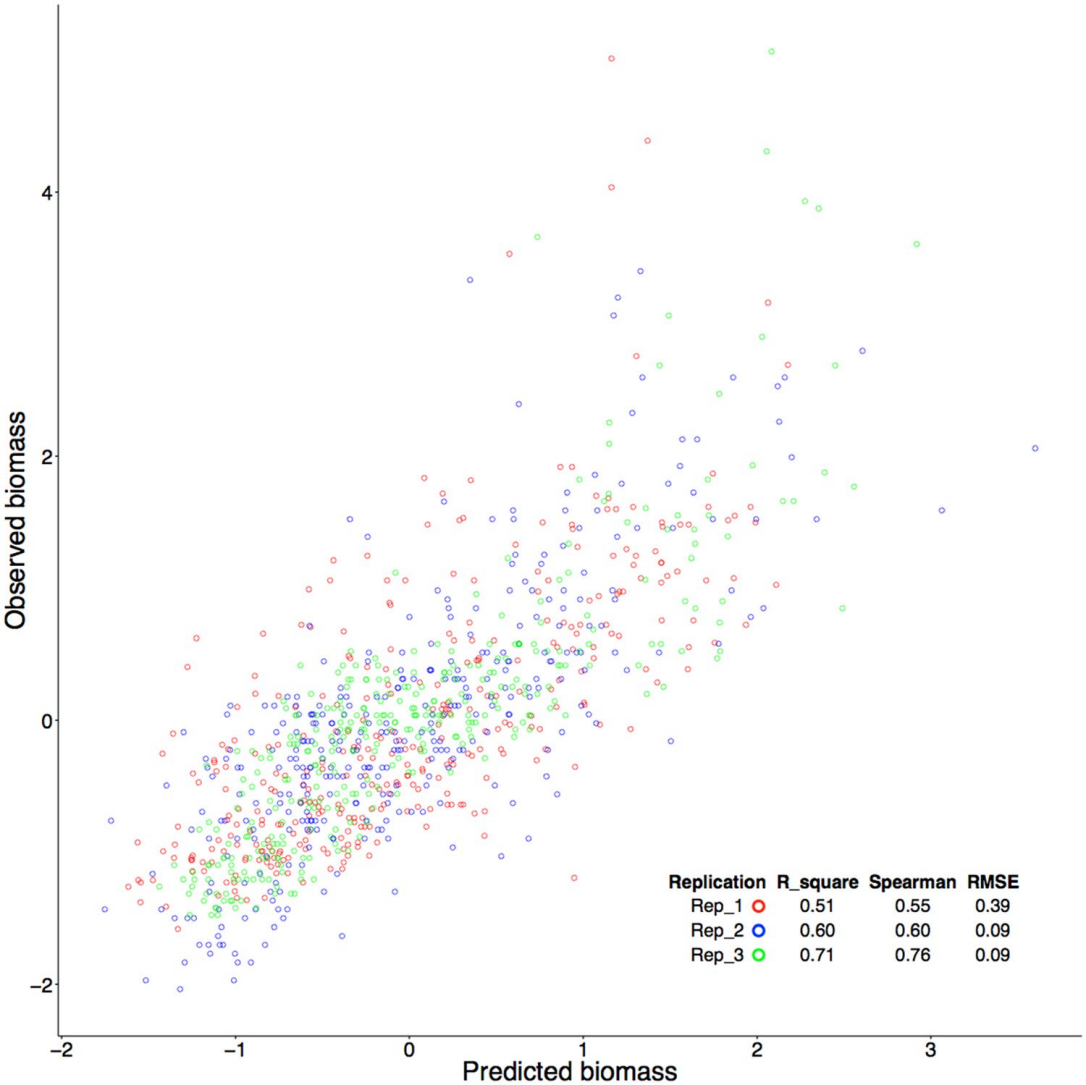
Validation of prediction accuracy in an independent field. The prediction model developed in the first field that was phenotyped and imaged in May, July, and September of 2019 was used to predict the biomass in the second field that was phenotyped and imaged in September of 2019. The three replicates in the second field are displayed with different colors. Prediction accuracy was calculated separately for each of the three replicates as the coefficient of determination (R^2^), Spearman correlation coefficient, and Root mean square error (RMSE) (kg).

The biomass in the second alfalfa field, planted in 2017, was not involved in the analyses until the comparisons between observed and predicted biomass. The predictions for this field (Fig. S6) were based on UAV images and the prediction formula derived from the first field. The prediction accuracies were relatively uniform, with R^2^ ranging from 0.51 to 0.71 among the three replicates (Fig. 5). The median R^2^ was 0.60. The Root Mean Square Error (RMSE) was calculated separately for each of the three replicates (1–3) as 0.39, 0.09, and 0.09 (kg), respectively. The validated results on the second field suggest that UAV images can potentially replace manual harvesting for biomass phenotyping. The manual harvesting took two days with five people. The UAV imaging took one person 10 min—about 500 times faster.

## Discussion

Both plant height and vegetative area were positively correlated with biomass. Although volume (height × area) was also positively correlated with biomass (R = 0.66), volume alone did not explain much error variation when area and height were included in the model (Fig. S9). We suggest two potential reasons. First, plant height and area were positively correlated. Second, we found that volume variation was mainly caused by the area of taller plots (Figs. S2, S4). The correlation between volume and height was stronger for shorter plots and weaker for taller plots. In contrast, we did not observe the same phenomena for the relationship between volume and area. The magnitude of correlation stayed the same regardless of whether plot areas were small or large. This result suggests that variation in volume for the taller plots was mainly controlled by area, not height.

For the first experimental field, we used all data to examine all correlations between biomass and 22 image features. Of the 22 features evaluated, canopy area, plant height, NDRE and NGRDI exhibited the highest correlations with biomass and the lowest correlations among each other. Thus, these four features were chosen to predict biomass. In doing so, we violated the rule of not using test data to develop a prediction model. However, because only four features were chosen compared to the 2424 observations, we assumed that the model overfitting problem should be minimal. This assumption was supported by the independent validation experiment with the second field, in which we used no observations to develop the prediction model.

We used the second field as an independent validation of the prediction from the first field. We only harvested and measured biomass of the second field once (September). Three analyses were performed. First, we displayed observed biomass as values (kg) and as color-coded heatmaps. Second, we compared the predicted biomass to the observed biomass by using all the image channels, including NIR and red edge. Third, we compared the predicted biomass with observed biomass. These analyses are shown in Figs. S1–S5. This process ensured the independence of the second field for validation. We did not use any information or data from the observed biomass values of the second field for training. The majority of biomass variation (R^2^ = 50–70%) in the test field was explained by the prediction model using UAV images. The analytical results not only demonstrate the feasibility of using UAV-based biomass phenotyping to substitute for manual phenotyping, but also revealed the need for phenotyping over multiple replicates and multiple harvest dates.

Among all replicate-month combinations for the first field, replicate 3 in September exhibited the lowest correlation coefficient (R = 0.31) between observed biomass and vegetative area calculated from UAV images. The remaining replicate-month combinations exhibited Pearson correlation coefficients above 0.71. During harvesting of replicate 3 on September 4, 2019, we experienced a rain event. Delaying the harvest was not an option because the plants would change dramatically after the rain. Therefore, we decided to continue harvesting during the rain. We suspect that the harvesting complications due to this unpredictable rain event resulted in biomass measurement errors for replicate 3-September and, in turn, likely caused the low prediction accuracies. For example, all prediction accuracies (R^2^) were below 0.1, compared to 0.5 and above for the other replicate-month combinations. Because UAV imaging can be completed quickly (e.g., ours took 10 min), such a weather problem can be avoided under real phenotyping situations.

Many unexpected factors can influence field experiments. For example, irrigation was a major environmental factor that influenced the amount of biomass in our experiment. UAV images helped identify two cases that could be misleading if these factors are not incorporated. First, the irrigation artifact contributed to the high correlations (e.g., R = 0.79 in replicate 1) between July and September. UAV images identified plot subgroups influenced by unintentional irrigation differences. As indicated by the degree of “greenness” in the UAV images across harvest dates (Fig. S1), the right three columns in replicate 1 and the top five rows of replicate 2 likely received more water than average. After removing these plots, the Pearson correlation coefficients (R) for biomass in the July and September harvests dropped to 0.65 and 0.55 for replicates 1 and 2, respectively.

The second case involved the identification of a potential cause for the outliers. UAV images visually demonstrated that the differences in biomass in the right three columns of plots in replicate 1 in September were likely created by additional irrigation. The standard deviation (kg) ranged from 0.55 to 0.62 among all replicates in May and from 0.74 to 0.91 in July. In September, the standard deviation was 0.11 and 0.15 for replicates 2 and 3, respectively. In contrast, the standard deviation was 0.35 for replicate 1, more than three times greater. After removing plots from the right three columns in replicate 1, the standard deviation became 0.12, similar to replicate 2 (0.11) and replicate 3 (0.15).

For most biomass estimation research using UAV images, Ground Control Points (GCPs) are applied to height calibration^28,30,31^. However, the measurement of GCPs is laborious and the equipment is expensive. Sometimes field conditions will also limit the application of GCPs^29^. When comparing the plant height and biomass in our study (Figs. S3–S5), the pattern of plant height roughly matched the change in biomass, meaning the estimation accuracy of relative plant height was acceptable even without GCPs. One reason for this result is our use of the SfM algorithm with highly overlapping images in the Pix4D software. Another probable reason is the fairly flat terrain of our study sites. Xiongzhe et al. also found plant height measured directly from UAV images without GCPs was highly correlated with ground truth measurements (R^2^ > 0.80)^38^.

The prediction accuracy in the independent field (the second) ranged from 0.51 to 0.71 (R^2^) among the three replicates, lower than two existing studies by Feng et al.^39^ (R^2^ = 0.87) and Cazenave et al. (R^2^ > 0.8)^33^. Although one distinction between our study and these other two is that our prediction accuracy was evaluated on an independent experimental field, other factors may have caused the accuracy differences. The study by Feng et al. involved two measurements at different times with dramatically different means. The plots of observed and predicted biomass exhibited two obvious clusters. The predicted biomass exhibited a much weaker correlation to the separate within-cluster observations compared to the correlation when clusters were combined. The study by Cazenave et al. used plots with ten individuals at most in each. Although a majority of the plots included 10 individuals, a substantial number of plots had only one individual. These two extremes can mathematically inflate the actual correlation values. Nevertheless, the prediction accuracies achieved in these studies are still promising for supporting high-throughput automatic phenotyping based on UAV imaging as a substitute the manual labor phenotyping.

## Conclusion

Genetic improvement in alfalfa biomass is in high demand. To achieve faster genetic improvements, we need high-selection intensity with faster and lower-cost phenotyping technology. In this study, we developed a fully automatic segmentation pipeline to extract features of alfalfa plots from UAV images, which were captured one day before harvest. A regression model with four features (canopy area, plant height, NDRE and NGRDI) achieved an accuracy of 0.5–0.7 (R^2^) for predicting plot biomass. The results suggest that high-throughput phenotyping with automatic plot segmentation of UAV images can be an efficient method to measure alfalfa biomass within field-plot settings and a low-cost substitution for the current labor-intensive, manual harvesting method. Importantly, widespread use of this new method promises to significantly contribute to faster genetic improvements in alfalfa breeding.

## Materials and methods

### Plant material and field management

Two fields in Prosser, Washington, were used for this study. The first and the second field were planted with a panel of germplasm composed of 212 alfalfa accessions in April of 2018 and 2017, respectively. A completely randomized block design with three replicates was used. Each replicate in the first field had 276 plots in total, arranged in 23 rows and 12 columns. Each plot was 1.52 m in length by 0.30 m in width. The distance between columns was 0.30 m; the distance between rows was 0.91 m. The dimensions of the whole first field measured 100 m × 60 m. Five rows (3rd, 8th, 13th, 18th, and 23rd row from the north) in each replicate were planted with a common accession as control checks. Each replicate in the second field had 23 rows and 15 columns. Four plots were left blank in each replicate. After removing blank plots, total number of plots equaled 808 and 1025 for the first field (Fig. S1) and the second field (Fig. S2), respectively.

Weeds were removed manually, and no other cover crop was planted in the fields. After the first cutting in May, irrigation was minimally applied at various intervals to ensure alfalfa plants survived the study site’s typical dry season (June to September). The timing of irrigation events was based on the field manager’s observations and judgement. In 2019, three harvest cuts were conducted on May 6–7, July 8–9, and September 3–4 for the first field, and one cut was conducted on September 3–4 for the second field. After cutting, fresh plant biomass was weighed for each individual plot.

### Unmanned aerial vehicle (UAV)-based remote sensing imagery

**Figure 6:**
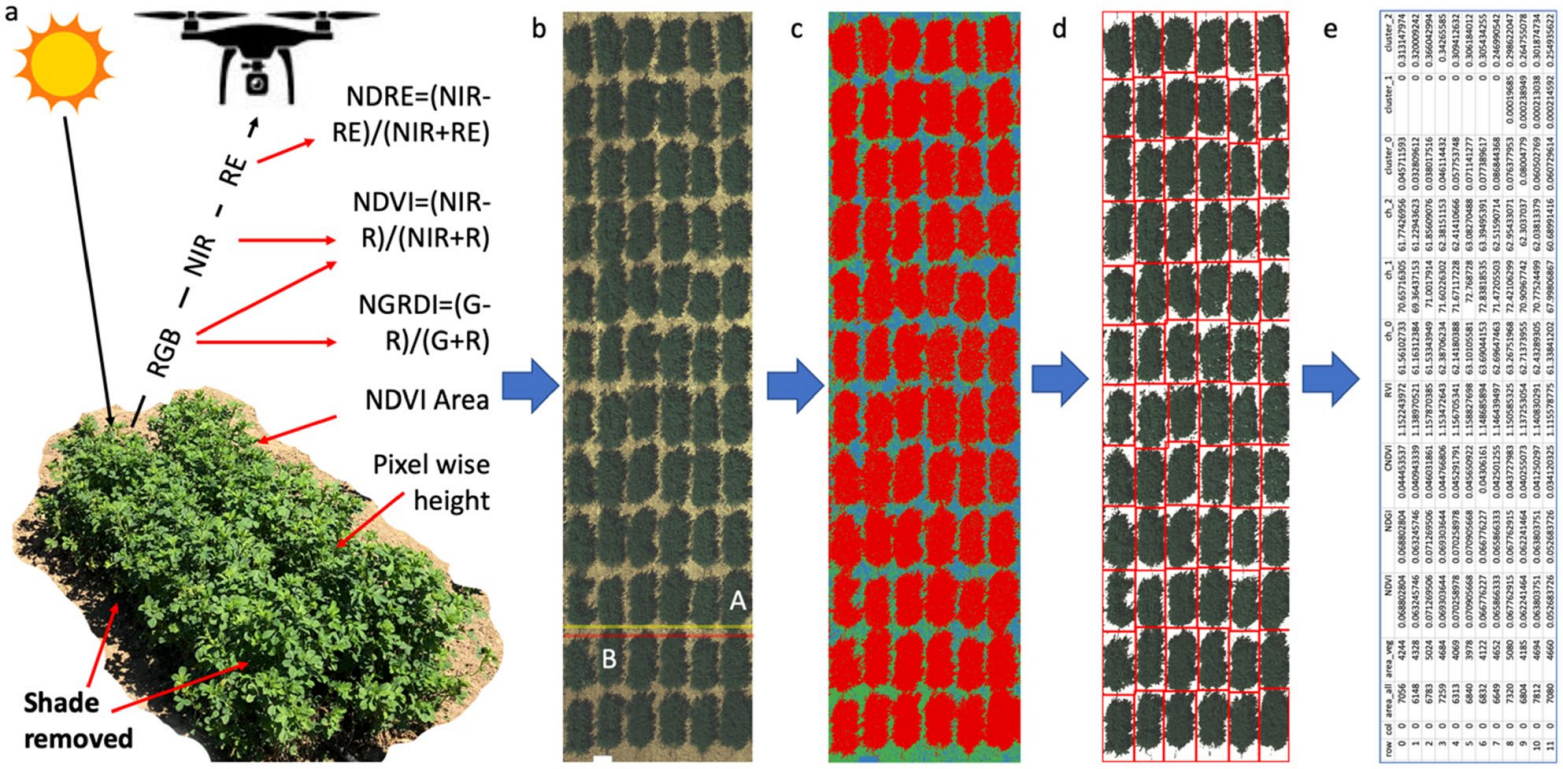
High-throughput phenotyping alfalfa biomass using UAV images. (**a**) The UAV-based imagery consists of five bands from the multispectral camera and pixel-wise plant height from the Pix4D, which can derive multiple Vegetation Indices (VIs) such as NDRE, NDVI, and NGRDI. The images with multiple bands are displayed as RGB (**b**). NDVI was used to cluster pixels into three classes (**c**). The class corresponding to the vegetation area (red) is selected to define pixels of interest and displayed in RGB (**d**). The plot information segmented by GRID is displayed for the plots in the first column (**e**). GRID can correctly segment plots that are not applicable using georeferences. For example, plot A and B cannot be separated by red and yellow lines, or any horizontal lines between them (**b**).

UAV imaging was conducted one day before each harvest in sunny weather (Fig. 6a). The time period between 11 a.m. and 3 p.m. was chosen for imaging to minimize sunshade. Remotely sensed data were collected with a multirotor DJI Phantom 4 Pro UAV (SZ DJI Technology Co., Shenzhen, China) equipped with a Double 4 K Multi-Spectral Ag camera (Sentera Inc., Minneapolis, USA). The camera was mounted with a 12.3 megapixels (3000 × 4100) Sony Exmor R IMX377 sensor, which can capture 5 spectral bands. The central wavelengths for blue, green, red, red edge, and NIR are 446 nm, 548 nm, 650 nm, 720 nm, and 840 nm, respectively, with full width at half maximum (FWHM) values of 40 nm, 45 nm, 70 nm, 40 nm, and 20 nm separately. A 15 cm × 15 cm white reference panel (MicaSense Inc., Seattle, USA) with a 60% nominal reflectance was used for the radiometric correction.

To ensure correct image acquisition during the flight, the FieldAgent Mobile App from Sentera was used to plan the flight path and automate the operation of the UAV. A constant flight height was maintained at 30.48 m and flight speed was fixed at 2.68 m/s. The ground sampling distance of imagery was 0.98 cm/pixel and an 80% overlap between two images was implemented. In total, about 260 images were taken for each harvest and stitched into a single image for analysis. Scanning the field of three replicates, approximately 6000 m^2^ in size, took about 10 min. The camera took about 100 images over the entire field.

### Image processing

The Pix4D mapper (version 4.1 from PIX4D, Prilly, Switzerland) was used to create the orthomosaic photo, digital surface model (DSM), and digital terrain model (DTM) with imagery from each flight. The pictures of the white reference panel recorded the reflectance condition of one specific flight, which enables the Pix4D to correct the pixel digital number values into absolute reflectance values. This calibration was realized automatically in Pix4D mapper by providing the images that captured the reference panel.

Point clouds were generated using structure from motion (SfM) algorithms in Pix4D mapper. Then, the DSM was produced with a dense point cloud. In Pix4D mapper, these photogrammetric 3D point data could be classified into vegetation, terrain, buildings, and other object categories based on color and geometric information and then output as a DTM^40^. The DTM is usually used to describe a terrain surface, whereas the DSM is used to represent surface elevation, including objects like plants and buildings on the ground. With the orthomosaic imagery from Pix4Dmapper, the QGIS2.18 (https://qgis.org/en/site/) was used to perform "raster calculate" and "raster bands merge". In QGIS, the raster calculator was used to subtract the DTM from the DSM to obtain the relative alfalfa canopy height^29^. Then, the GDAL toolbox in QGIS was implemented to organize visible band (R, G, and B), invisible band (red edge and near-infrared), and canopy height raster images into a single raster image with six layers. The merged file was saved as a TIFF format with a size of about 1 GB for the entire field.

### Plot segmentation and image feature extraction

Instead of manually drawing the boundary of plant plots on the aerial images, we used GRID^37^ to help extract pixels of interest from each plot automatically. GRID provides an accessible graphical user interface (GUI) to implement plot extraction. By applying k-means cluster analysis, users can define the cluster numbers with any band of raster image. By default, all pixels in a raster image are classified into three clusters (vegetation, soil, and the rest) with the red and near-infrared channels. In our case, for UAV images of all replicates and all months, the first class corresponded to pixels comprised of plants, the second class corresponded to pixels of shade or drainage pipe, and the third class corresponded to pixels of bare soil (Fig. 6b,c). The plant class was used to extract features for each plot (Fig. 6d), including total number pixels of plant area, average of original channels, average Normalized Difference Red Edge index (NDRE) and Normalized Green–Red Difference Index (NGRDI), and pixel-wise average height in the plant area (Fig. 6e).

After defining pixels of interest, the grid pattern of a plot can be detected automatically in GRID. More details can be found in the GRID website (http://zzlab.net/GRID). With the help of the alfalfa field plant map, GRID can assign each variety number to pixels extracted from each plot. Finally, GRID can output the average value of each spectral band for each alfalfa plot, with which we can calculate different vegetation indices (VIs) for each alfalfa plot. The relationships among the original UAV image channels and plant height across plots is displayed as heatmaps and scatter plots (Figs. S10–S11) with R (https://www.R-project.org/).

### Biomass modeling

The first field data were used as training data and the second field data were used as testing data. The training data were from three different months and three plant experimental fields. Therefore, data were standardized within each month and replication location to remove the various month and territory effects in the three replications before modeling biomass. After standardization, the average and the standard deviation of each replication within each month were assigned 0 and 1, respectively. All 22 image features, including plant area, plant height, volume, and 19 VIs (Table S1), were evaluated. Their correlations with biomass are shown with Pearson correlation coefficients (R) in Fig. S7.

Two cluster analyses among these 22 features were implemented after standardization (Fig. 3) and before standardization (Fig. S8) with the "pheatmap" package in R (https://CRAN.R-project.org/package=pheatmap). One candidate variable is selected from each cluster for future modeling. To reduce the overfitting problem, the cluster including Normalized Difference Vegetation Index (NDVI) was excluded because NDVI had been used to define canopy area.

Additionally, we examined the contribution of each single factor. The residual sum of squares (RSS) from the full model predicting biomass with 22 features was treated as the base. Each variable was moved from the full model one by one and the RSS was recalculated. The increase in RSS was treated as the contribution of the specific feature moved out and shown in Fig. S9. The key function during this analysis was "lm" function in R. The feature in each cluster with highest contribution was included in future model. Ultimately, canopy area (defined by NDVI), plant average height, NDRE, and NGRDI from the GRID software were used to model alfalfa biomass in a multiple linear regression (MLR). Their correlations with biomass are shown in detail among each month and each replication in Fig. 2.

Then, we implemented a threefold cross-validation among the training data. We used three different ways to split training data, including spatial, temporal, and a mixture of spatial and temporal. First, data were divided into three parts by month to implement threefold cross-validation. Second, data were similarly split into the three parts based on replication to execute threefold cross-validation. Third, all the training data were split into 3 parts randomly across both month and replication to carry out the cross-validation. This random split process was repeated 100 times. During training, the coefficient of determination (R^2^) was used to evaluate accuracy. For testing of the second field data, the R^2^, spearman rank correlation coefficient, and root mean square error (RMSE) were used to evaluate accuracy. The unit of RMSE is kg. The biomass prediction equation obtained with all training data, after removing replicate 3 field data in September (outlier) is as follows:

1 $$ {\text{Biomass}}\;{\text{estimated}} = \, 0.70*{\text{Area}} + \, 0.07*{\text{Height}} + \, 0.18*{\text{NGRDI}} + \, 0.07*{\text{NDRE}} $$

## Supplementary Information

Supplementary Information.
