# Supplementary material for: Validation of UAV-based alfalfa biomass predictability using photogrammetry with fully automatic plot segmentation
Source: Sci Rep. 2021 Feb 8;11:3336. doi: 10.1038/s41598-021-82797-x (PMC7870825; doi:10.1038/s41598-021-82797-x)
Supplement: Supplementary file 1 — Supplementary Information. [file 41598_2021_82797_MOESM1_ESM.docx]

**Validation of UAV-Based Alfalfa Biomass Predictability Using Photogrammetry with Fully Automatic Plot Segmentation**

Department of Crop and Soil Sciences, Washington State University, Pullman, Washington State, 99163, USA

**Zhiwu Zhang:**

**PhD, WGC Distinguished Professorship for Statistical Genomics (zhiwu.zhang@wsu.edu)**

**Website (Personal):** [**http://css.wsu.edu/zhiwu-zhang**](http://css.wsu.edu/zhiwu-zhang)

**Website (Lab):** [**http://zzlab.net**](http://zzlab.net)

**Zhou Tang:**

**PhD student (zhou.tang@wsu.edu)**

**Atit Parajuli:**

**PhD student (atit.parajuli@wsu.edu)**

**Chunpeng James Chen:**

**PhD student (chun-peng.chen@wsu.edu)**

**Yang Hu:**

**PhD, Post-doctoral researcher (yang.hu@wsu.edu)**

**Samuel Revolinski:**

**PhD student (samuel.revolinski@wsu.edu)**

United States Department of Agriculture-Agricultural Research Service, Plant Germplasm Introduction and Testing Research, 24106 N Bunn Road, Prosser, Washington State, 99350 USA

**Long-Xi Yu:**

**Research Geneticist (Plants) (**[**Longxi.Yu@ars.usda.gov**](mailto:Longxi.Yu@ars.usda.gov)**)**

**Cesar Augusto Medina:**

**PhD student (cesar.medinaculma@wsu.edu)**

**Sen Lin:**

**PhD student (sen.lin@wsu.edu)**

**Supplementary**


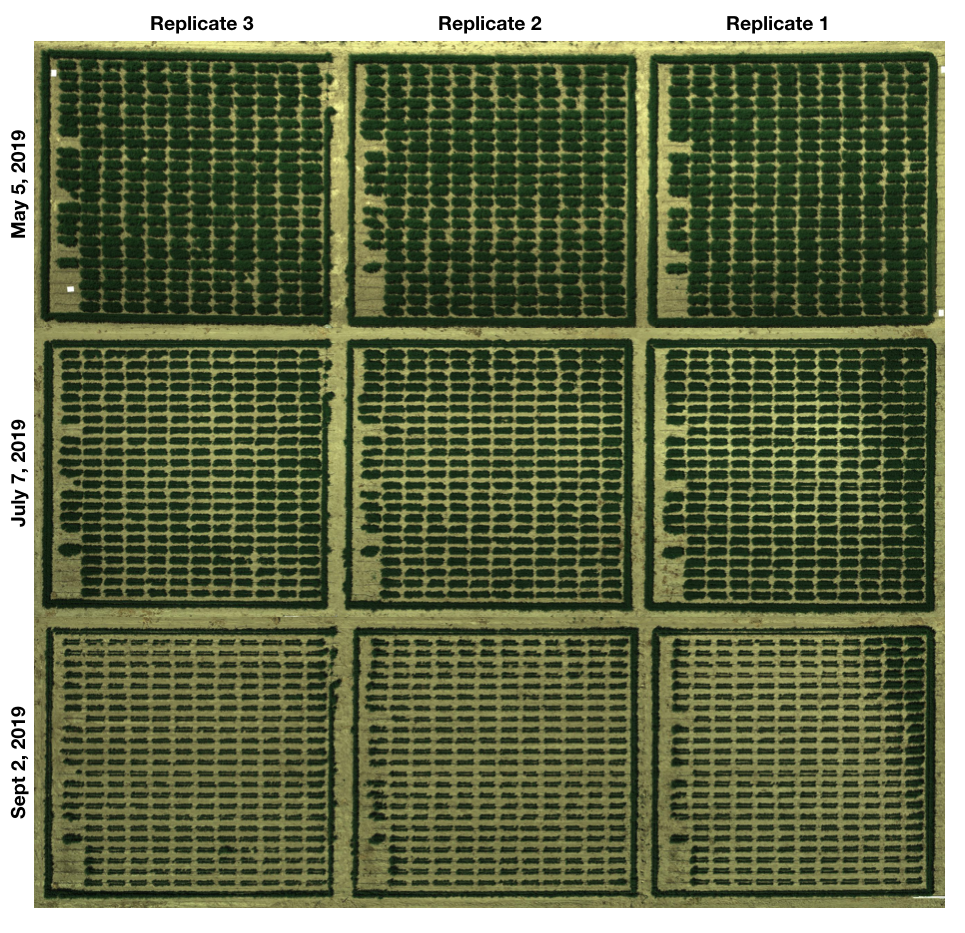


**Figure S1. Stitched UAV images of the first alfalfa field with three replicates.** The raw images were taken at 30.48 m with 80% overlap. Pix4D mapping software was used to stitch the image and derive plant height. The images contain six channels: red, green, blue, near-infrared, red edge, and height. The RGB images are displayed with replicates as columns and months of harvest as rows.


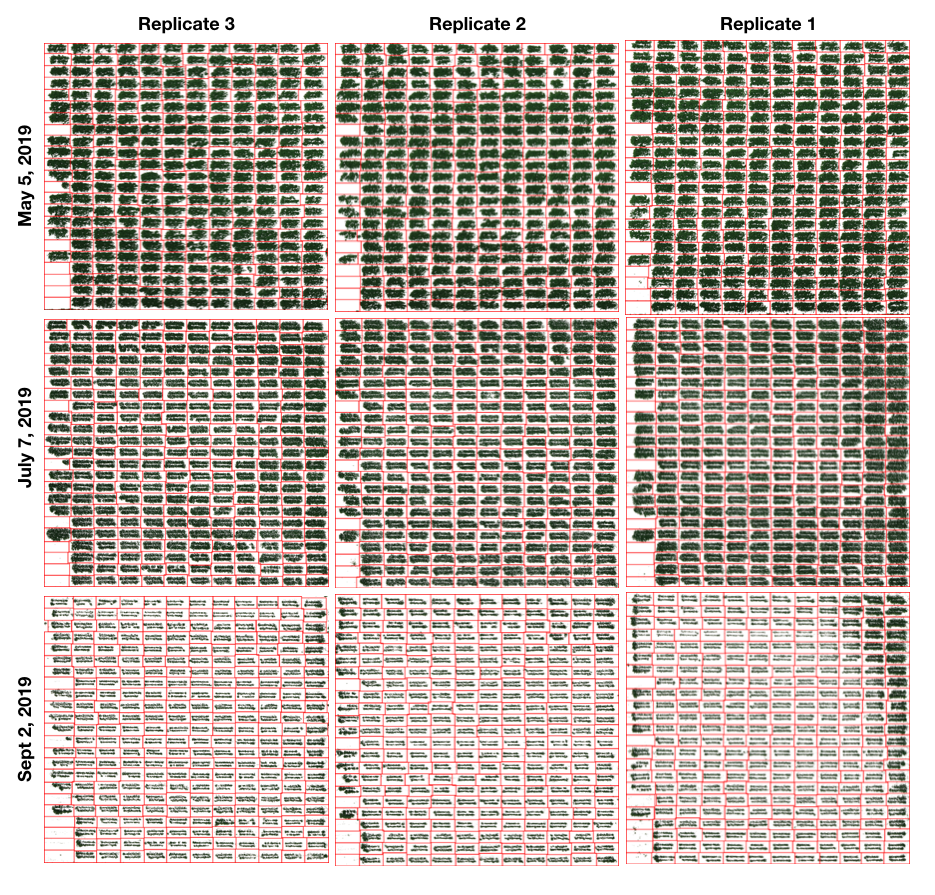


**Figure S2. Segmentation of UAV images of the first alfalfa field.** The segmentation was based on NDVI with three clusters. The extracted pixels of interest are displayed as RGB images with replicates as columns and months of harvest as rows.


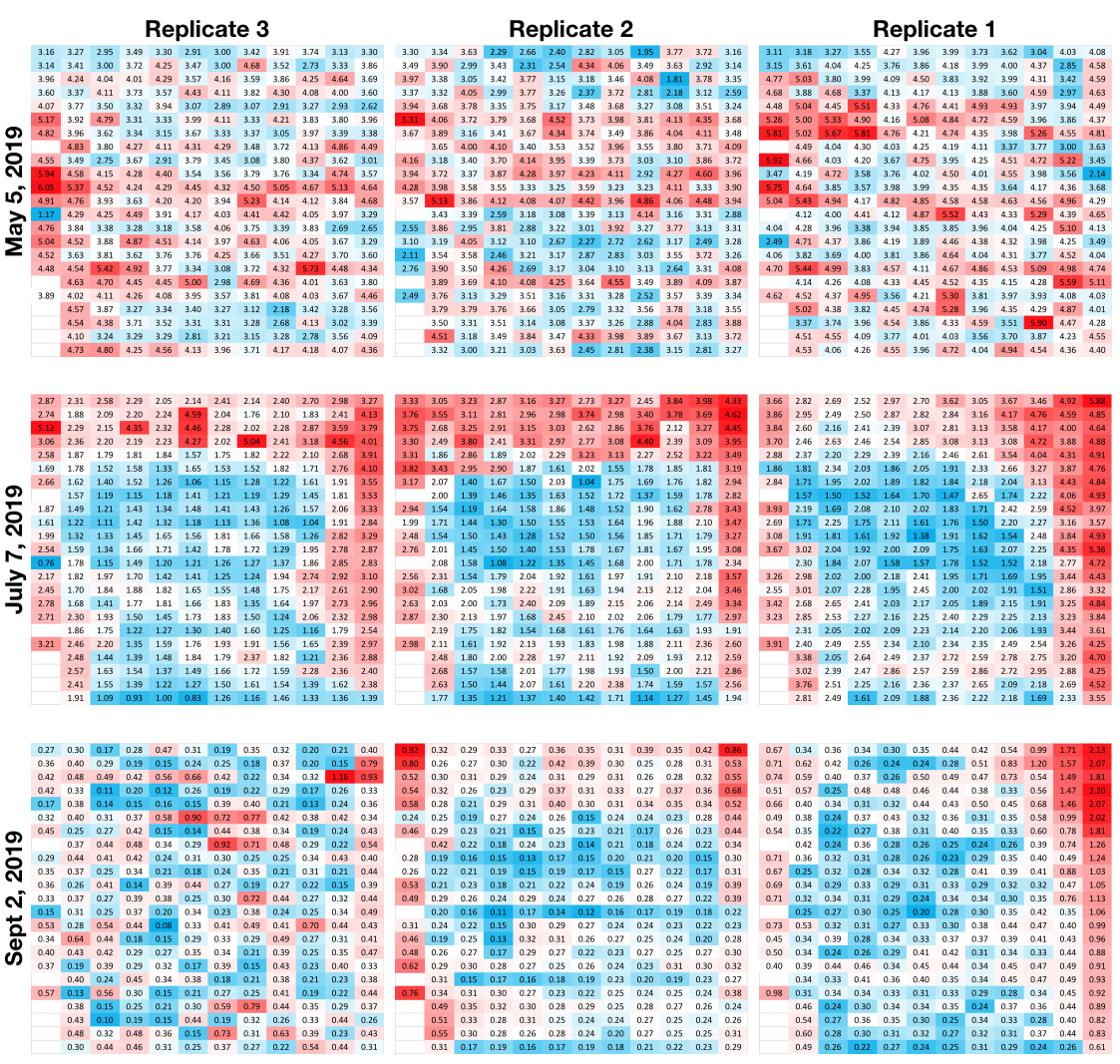


**Figure S3. Observed biomass in the first alfalfa field.** Biomass is displayed as both measured values (kg) and color-coded heatmap values. The heatmaps were coded for each plot in each month-replicate combination, ranging from dark red (highest biomass) to dark blue (lowest biomass), with months of harvest as rows and replicates as columns.

**
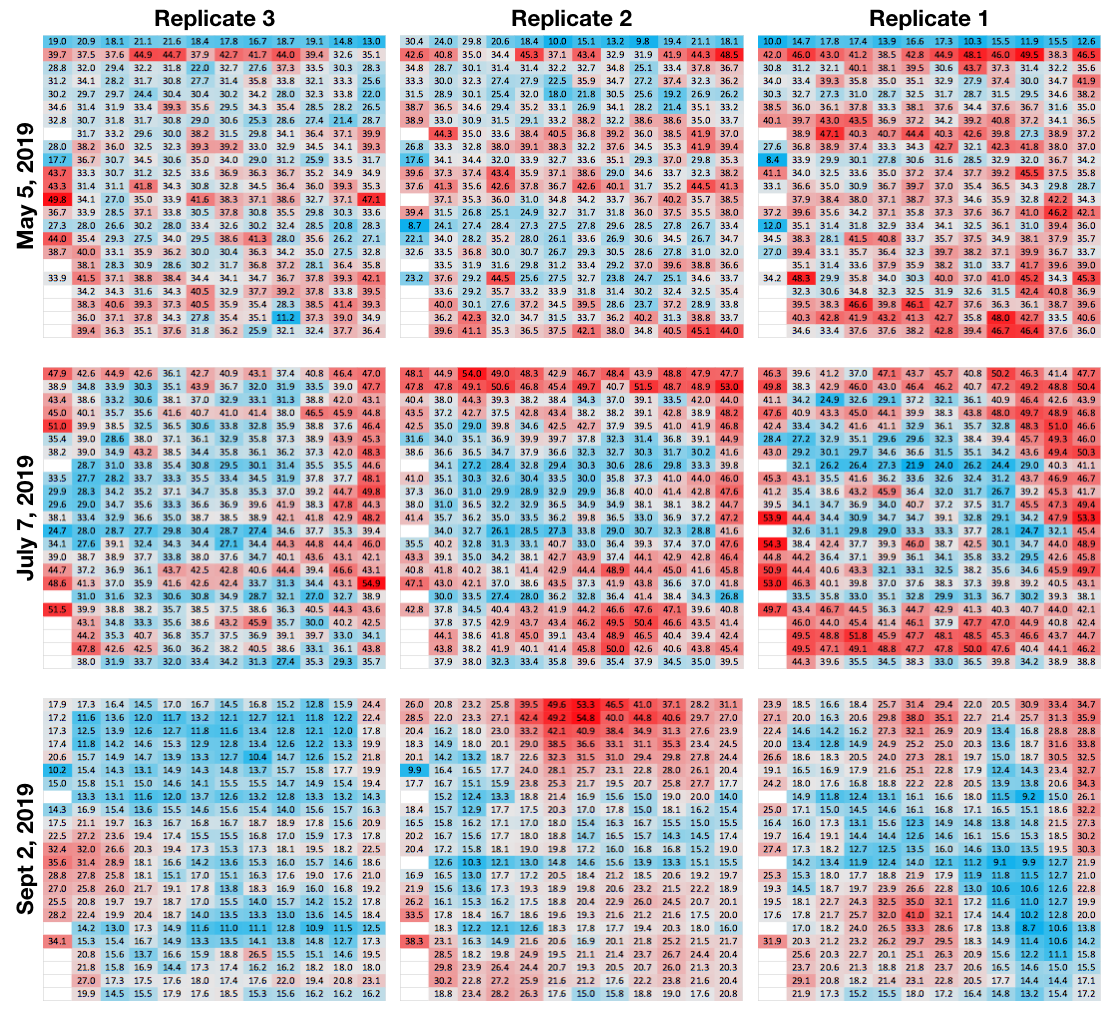
**

**Figure S4. Plant height in the first alfalfa field.** Average alfalfa plant heights for each plot in each month-replicate combination are displayed as both calculated values (cm) and color-coded heatmap values. Dark red represents the tallest plots and dark blue the shortest, with harvest months as rows and replicates as columns.

**
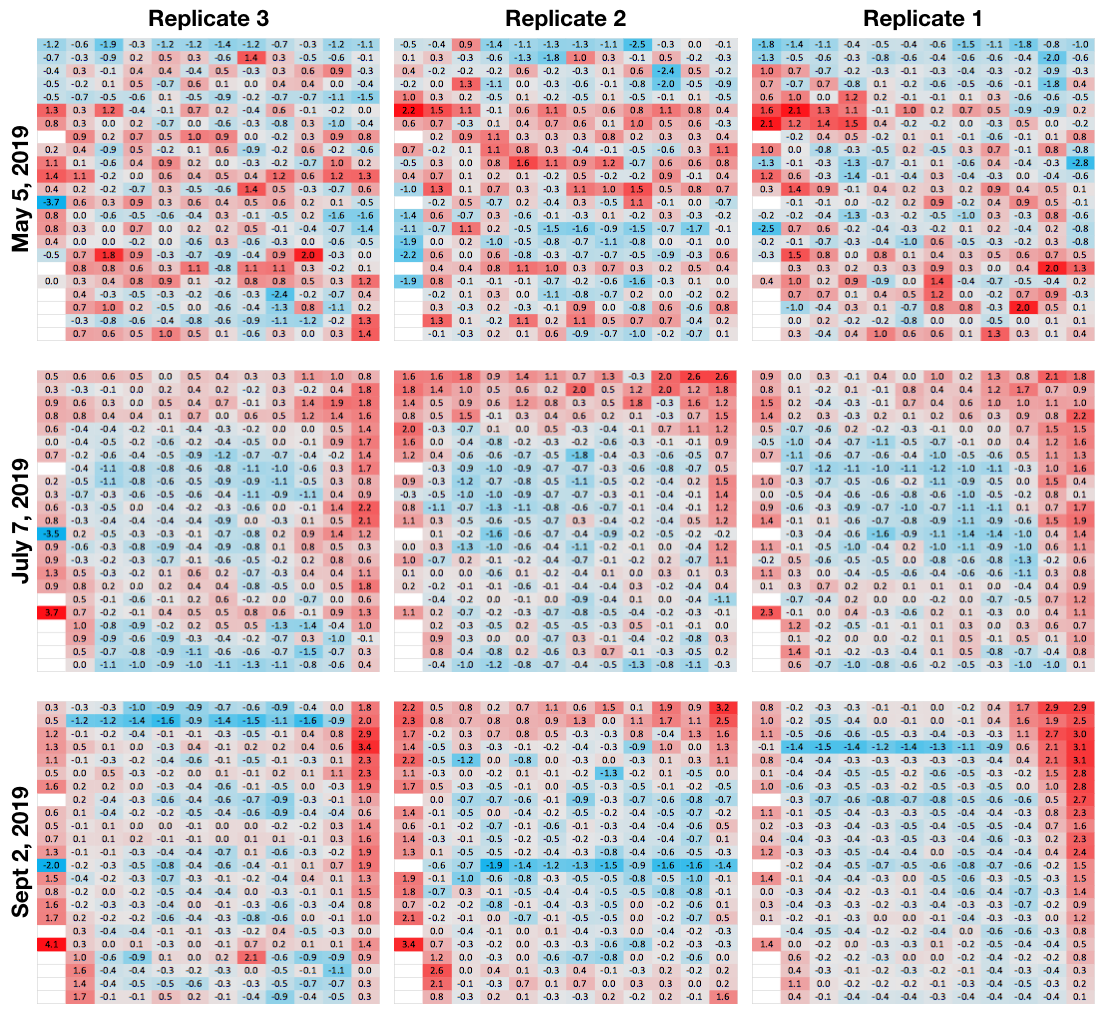
**

**Figure S5. Predicted biomass of the first alfalfa field.** Predicted biomass is displayed for each plot as both predicted values (kg) and color-coded heatmap values (dark red represents the highest biomass and dark blue the lowest biomass), with months as rows and replicates as columns.

**
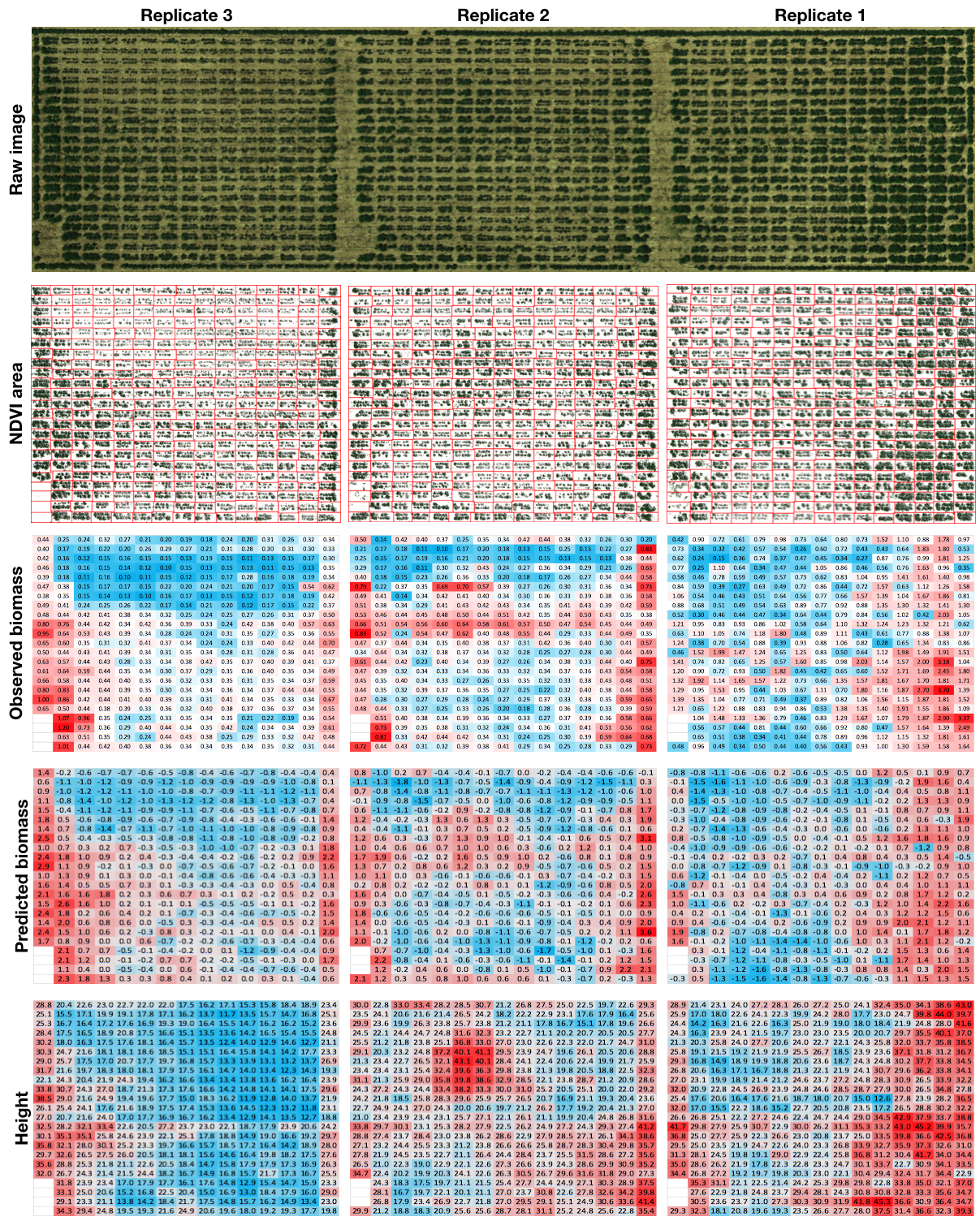
**

**Figure S6. Validation of biomass prediction in the second alfalfa field.** The second field contains three replications displayed as columns. The field was imaged one day prior to harvesting on September 2, 2019. The raw images were taken at 30.48 m, with 80% overlap. Pix4D software was used to stitch the image and derive plant height. The images contain six channels: red, green, blue, near-infrared, red edge, and height. The RGB images are displayed in the top row, followed by the extracted pixels of interest displayed as red-green-blue panels in the next row, the heatmap of observed biomass (kg), the heatmap of predicted biomass, and the calculated height in the bottom row.

**
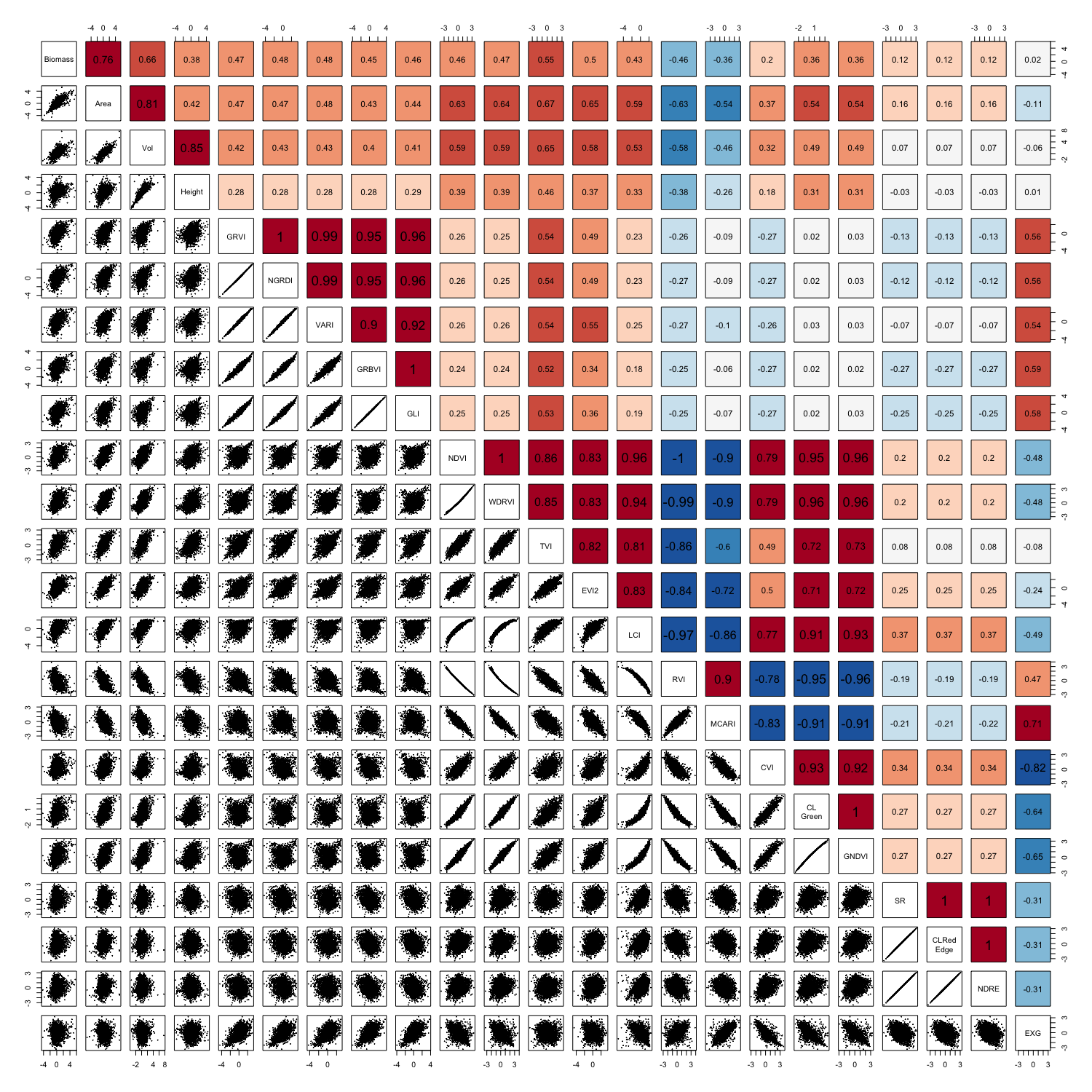
**

**Figure S7. Correlation among biomass and UAV image features.** The image features include plant area, height, volume, and 19 indices defined in Table S1. The correlations are demonstrated as Pearson correlation coefficients in the upper triangular area and scatter plots in the lower triangular area. Image features were standardized within combinations of month and replicate.

**
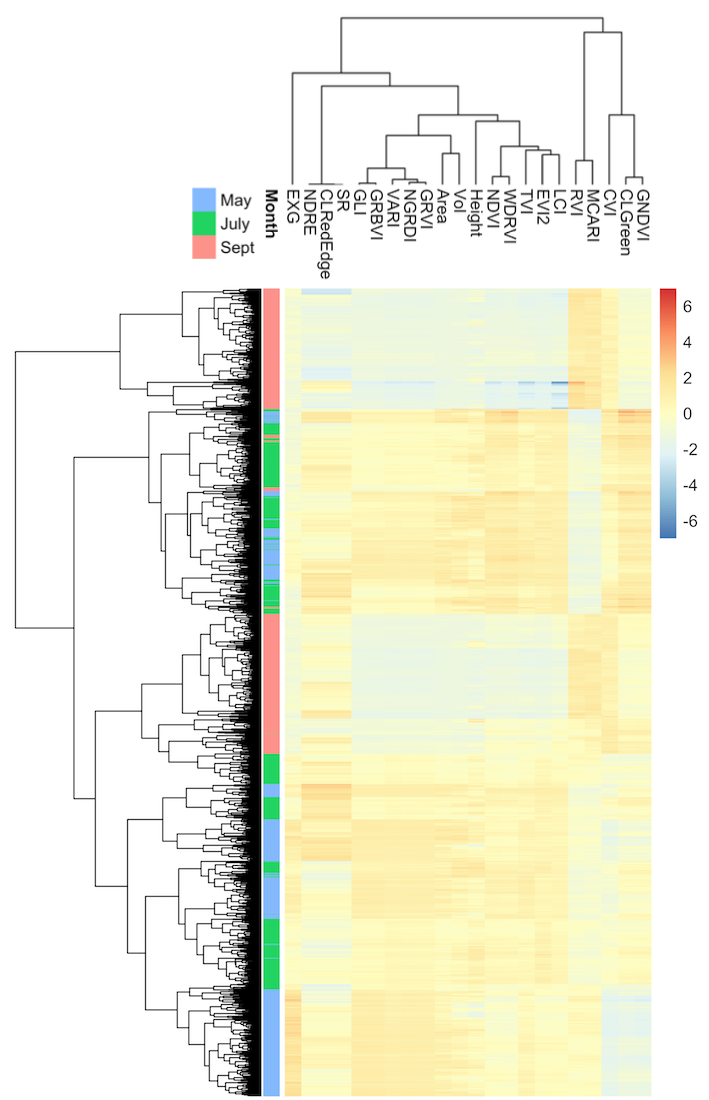
**

**Figure S8. Two-way cluster analysis of plots and image features before standardization.** The image features include plant height and 19 indices defined in Table S1.

**
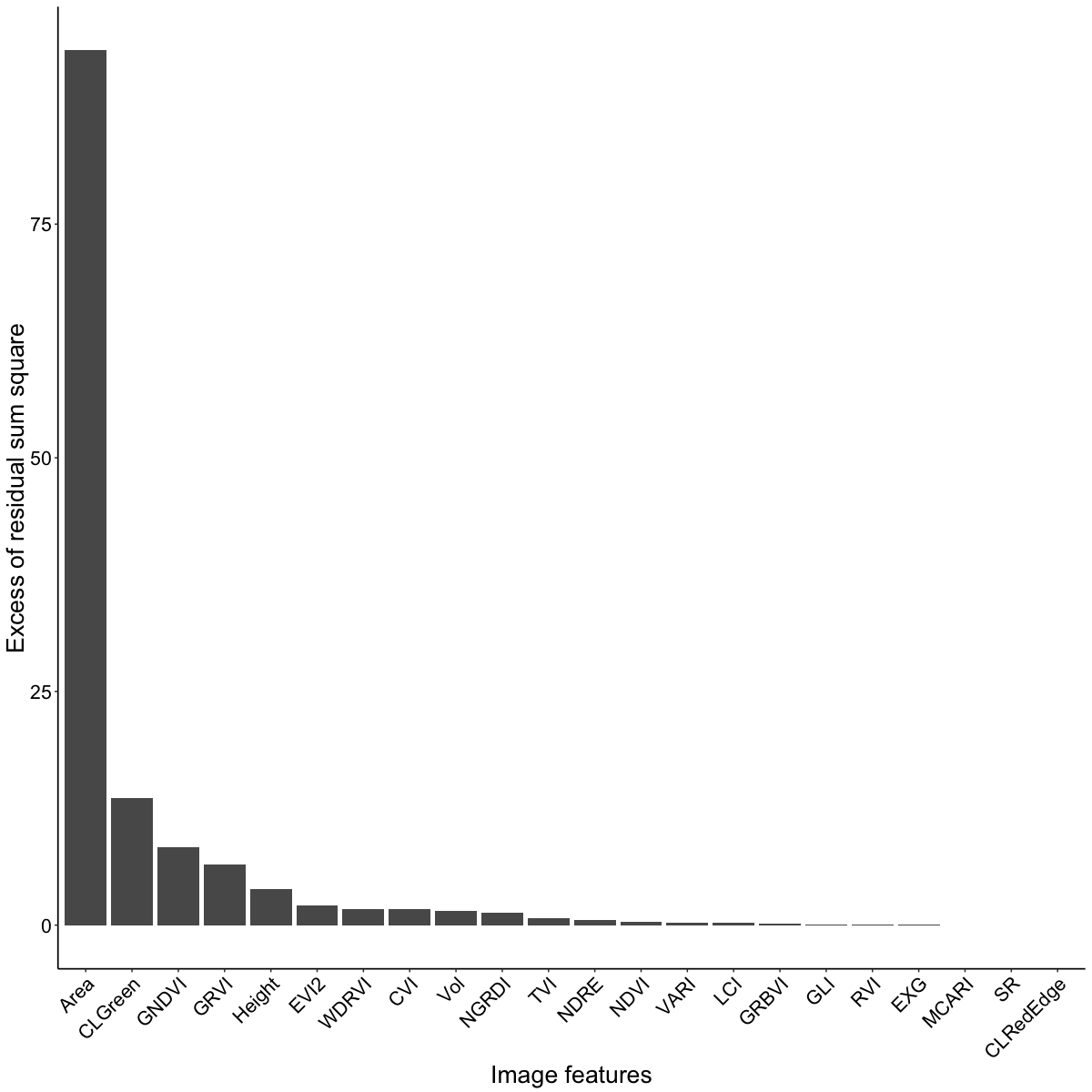
**

**Figure S9. Single feature contributions to biomass variation.** The base residual sum of squares (RSS) was from the full model, with all image features included as covariates. The contribution of a feature is presented as the excess, or increase in, the RSS after the feature was removed from the full model. The larger the excess, the greater the feature's contribution to explaining biomass variation.

.
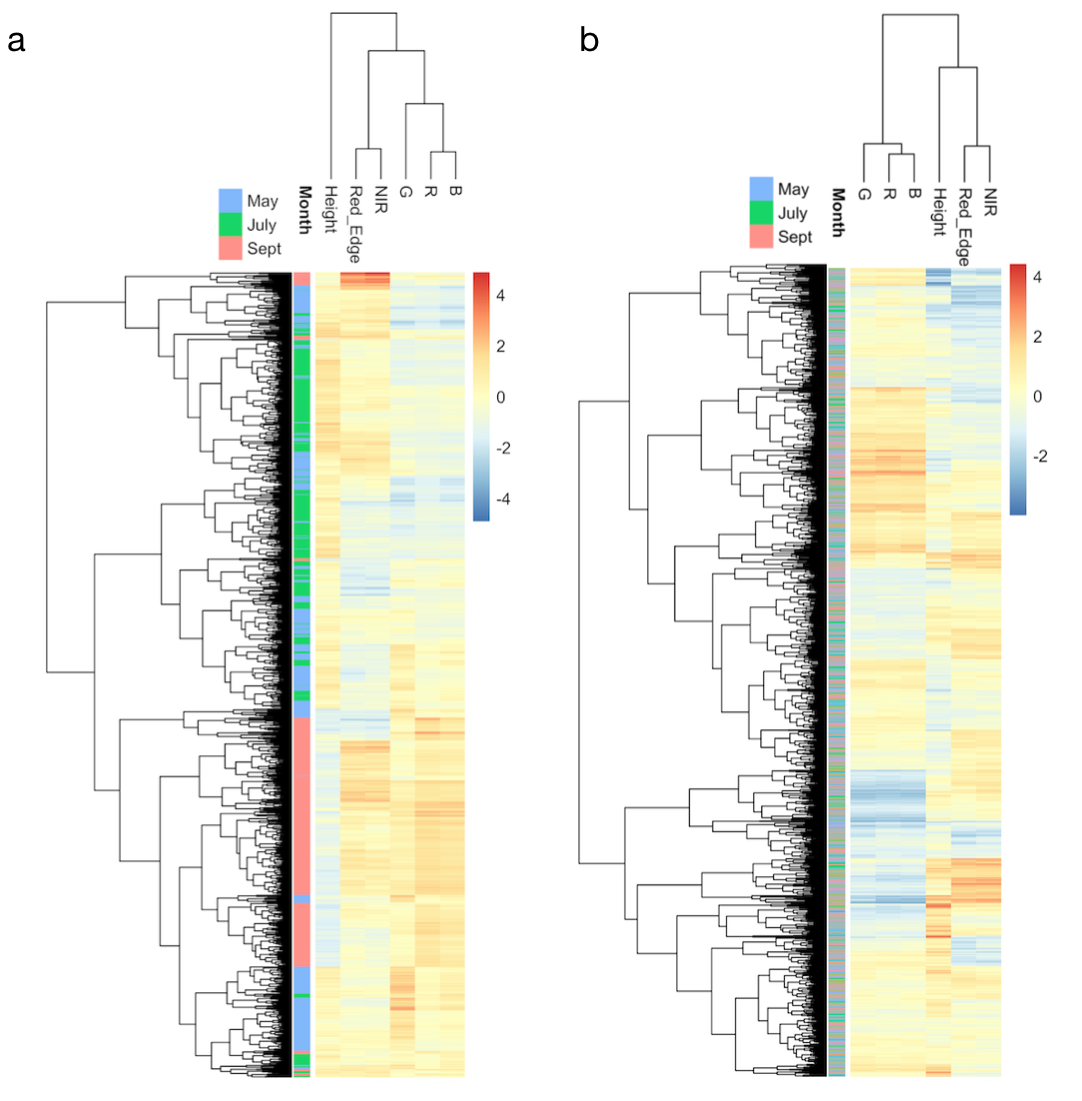


**Figure S10. Two-way cluster analysis of plots and UAV image channels and plant height.** The plots are displayed before (a) and after (b) standardization within each combination of month and replicate. The channels include visible R (Red), G (Green), and B (Blue) and non-visible NIR (Near-Infrared) and Red_Edge.

**
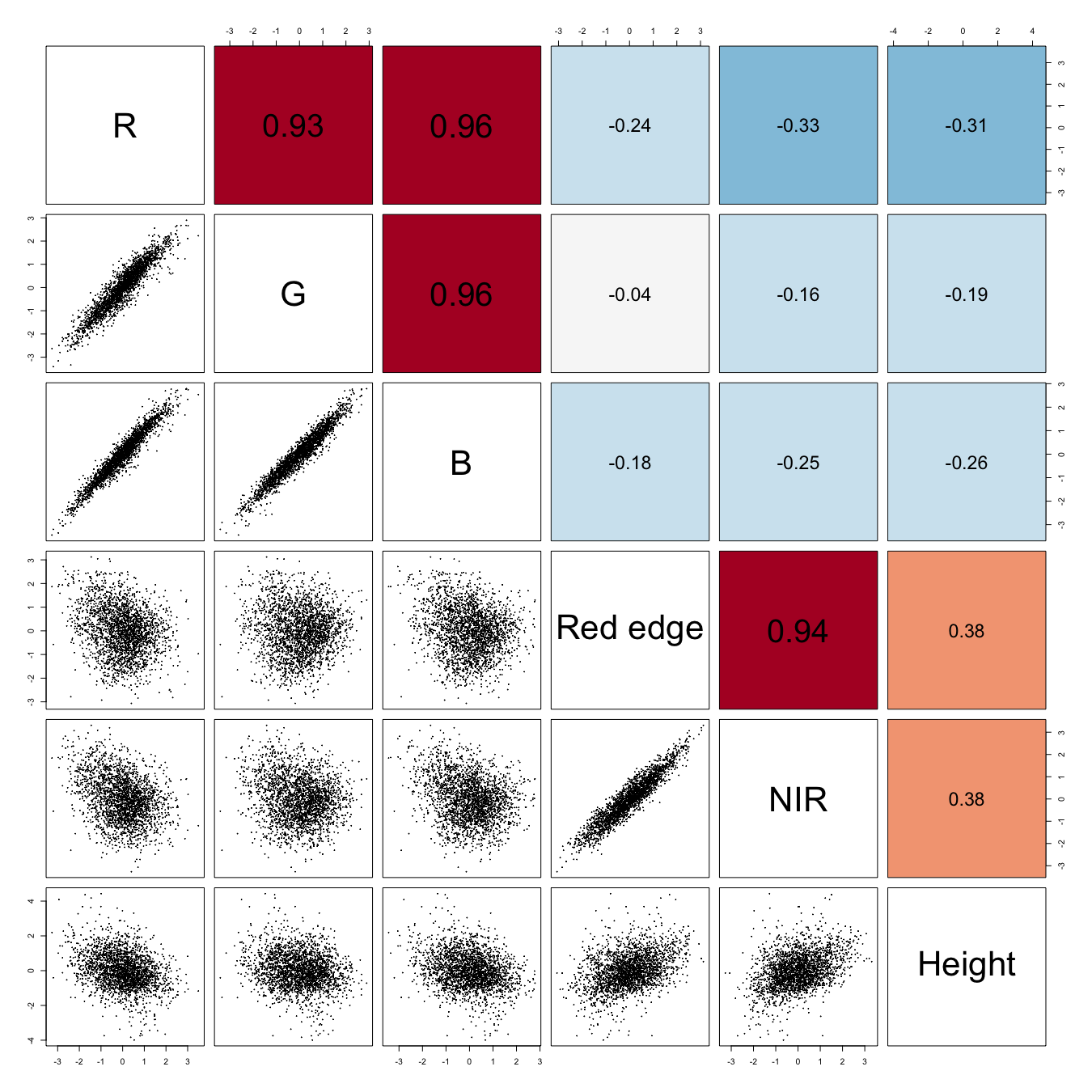
**

**Figure S11. Correlation among UAV image channels and plant height.** The channels include visible R (Red), G (Green), and B (Blue) and non-visible Red edge and NIR (Near-Infrared). The correlations are demonstrated as Pearson correlation coefficients in the upper triangular area and scatter plots in the lower triangular area. Image features were standardized within month-replicate combinations.

**Table S1. Indices derived from visible and non-visible channels.** Visible channels include R (Red), G (Green), and B (Blue) and non-visible channels include NIR (Near Infrared) and RE (Red Edge).

| **Index** | **Description** | **Formula** | **RGB only** |
| --- | --- | --- | --- |
| ExG | Excess Green Index | 2*G-R-B | Y |
| GLI | Green Leaf Index | (2*G-R-B)/(2*G+R+B) | Y |
| GRBVI | Green Red Blue Vegetation Index | (G^2 - B*R)/(G^2 + B*R) | Y |
| NGRDI | Normalized Green-Red Difference Index | (G-R)/(G+R) | Y |
| VARI | Visible Atmospherically Resistant Index | (G-R)/(G+R-B) | Y |
| CI_green_ | Chlorophyll index of Green | (N-G)/G | N |
| CI_red-edge_ | Chlorophyll index of Red Edge | (N-E)/E | N |
| CVI | Chlorophyll Vegetation Index | (N*R)/(G*G) | N |
| EVI2 | Enhanced Vegetation Index | 2.5*(N-R)/(N+6*R-7.5*B+1) | N |
| GNDVI | Green Normalized Difference Vegetation Index | (N-G)/(N+G) | N |
| GRVI | Green Ratio Vegetation Index | N/G | N |
| LCI | Leaf Chlorophyll Index | (N-E)/(N-R) | N |
| MCARI | Modified Chlorophyll Absorption in Reflectance Index | ((E - R)- 0.2*(E-G))*E/R | N |
| NDRE | Normalized Difference Red Edge Index | (N-E)/(N+E) | N |
| NDVI | Normalized Difference of Vegetation Index | (N-R)/(N+R) | N |
| RVI | Ratio Vegetation Index | R/N | N |
| SR | Simple Ratio | N/R | N |
| TVI | Triangular Vegetation Index | 60*(N-R)-100(R-G) | N |
| WDRVI | Wide Dynamic Range Vegetation Index | (0.1*N-R)/(0.1*N+R) | N |
